# Stem Cell Cytoskeletal Responses to Pulsatile Flow in Heart Valve Tissue Engineering Studies

**DOI:** 10.3389/fcvm.2018.00058

**Published:** 2018-06-05

**Authors:** Glenda Castellanos, Sana Nasim, Denise M. Almora, Sasmita Rath, Sharan Ramaswamy

**Affiliations:** Tissue Engineered Mechanics Imaging and Materials Laboratory, Biomedical Engineering, Florida International University, Miami, FL, United States

**Keywords:** pulsatile shear stress, HBMSCs, actin filaments, cytoskeleton, nuclear, tissue engineering, *klf2a*, heart valves

## Abstract

Heart valve replacement options remain exceedingly limited for pediatric patients because they cannot accommodate somatic growth. To overcome this shortcoming, heart valve tissue engineering using human bone marrow stem cells (HBMSCs) has been considered a potential solution to the treatment of critical congenital valvular defects. The mechanical environments during *in vitro* culture are key regulators of progenitor cell fate. Here, we report on alterations in HBMSCs, specifically in their actin cytoskeleton and their nucleus under fluid-induced shear stresses of relevance to heart valves. HBMSCs were seeded in microfluidic channels and were exposed to the following conditions: pulsatile shear stress (PSS), steady shear stress (SS), and no flow controls (*n* = 4/group). Changes to the actin filament structure were monitored and subsequent gene expression was evaluated. A significant increase (*p* < 0.05) in the number of actin filaments, filament density and angle (between 30° and 84°), and conversely a significant decrease (*p* < 0.05) in the length of the filaments were observed when the HBMSCs were exposed to PSS for 48 h compared to SS and no flow conditions. No significant differences in nuclear shape were observed among the groups (*p* > 0.05). Of particular relevance to valvulogenesis, *klf2a*, a critical gene in valve development, was significantly expressed only by the PSS group (*p* < 0.05). We conclude that HBMSCs respond to PSS by alterations to their actin filament structure that are distinct from SS and no flow conditions. These changes coupled with the subsequent gene expression findings suggest that at the cellular level, the immediate effect of PSS is to initiate a unique set of quantifiable cytoskeletal events (increased actin filament number, density and angle, but decrease in filament length) in stem cells, which could be useful in the fine-tuning of *in vitro* protocols in heart valve tissue engineering.

## Introduction

Congenital heart defects occur in four to six infants out of every 1,000 births (1). Approximately 24.5% of neonatal mortality is attributed to congenital heart defects (2). Among the plethora of cardiovascular defects, one of the more common, yet life-threatening conditions, is critical aortic valve stenosis (AVS), which results in high mortality and morbidity despite early interventions (3). The developmental mechanisms that lead to critical AVS are unknown. However, AVS is characterized by poor or missing valve commissures in fetal development, as well as abnormal leaflet fusion; infection, specifically endocarditis *in utero* has been proposed as one of the causal factors (4–9). The most severe forms of congenital heart disease have an incidence rate of ~20,000 live births/year (10), and of these, ~1/3 of cases present problems associated with the aortic heart valve.

Under normal circumstances, valve leaflet composition and structure permits endurance of demanding mechanical forces as they function under a complex, coupled loading state of cyclic tensile, cyclic flexure,-and fluid-induced shear stresses, including oscillatory shear stresses (PSS) (11–13). However, in AVS, significant systolic transvalvular pressure gradients with a mean > 60 mmHg, resulting from narrowing of the aortic root, imposes considerable workload on the left ventricle, leading to rapid heart failure if left untreated (14–16).

Over the last 20 years, tissue engineered heart valves (TEHVs) derived from stem cells have been investigated to overcome the shortcomings associated with treatment of critical valve anomalies in children (17–20). Recent studies have applied mechanical stimuli to formulate tissue structures with enhanced extracellular matrix (ECM) properties resembling native heart valves, in particular, using human bone marrow-derived stem cells (HBMSCs) seeded onto biodegradable scaffolds (18, 21). These mechanical stimuli are sensed by the cell membrane receptors, later transferred to the cytoskeleton; consequently, these stimuli initiate a biochemical signaling cascade (22). In HBMSCs, the cytoskeletal structure has shown to be altered after exposure to fluid-induced shear stress (23). The actin filaments of the cell cytoskeleton serve as structural contributors to modulation of subsequent cell biological responses, including gene expression, cellular, and ECM synthesis (24). In general, it has been shown that the application of mechanical stress on actin filaments causes cytoskeleton reorganization, leading to tissue remodeling affecting stem cell viability, self-renewal, and differentiation (25). Although, detailed characterization of intracellular structures, specifically in response to fluid-induced shear stress, is not known. In addition, other cellular components such as focal adhesions, integrin, and the nucleus collectively play important roles in modulating cellular biological responses (18).

Our laboratory has previously demonstrated that HBMSC-derived tissue formation and flow-responsive differential regulation is robust when cell-seeded constructs are cultured under fluid-induced PSS environments. Spatial distribution of HBMSCs that differentiated to the endothelial phenotype (CD31+) were largely found on the tissue surfaces, while cells with an activated myofibroblast phenotype (αSMA +) were mostly aggregated in the interstitial space, similar to the native heart valve cellular-makeup (12, 13, 26, 27). Specifically, PSS regulates HBMSCs structure and has been shown to be highly relevant to both native heart valve development and to TEHVs (27–29). Yet, identifying alterations in the HBMSCs cytoskeleton during PSS exposure may lead to a deeper understanding of specific changes at the cell structural-level. Our underlying hypothesis is that cytoskeletal changes, primarily with actin filaments can be quantified as they can be monitored during culture. Such quantification can serve as early indicators of *in vitro* differential HBMSCs regulation for functional TEHV development and optimization. Thus, in this investigation we applied PSS to growing HBMSCs to understand fundamental cellular structural responses that precede the resulting gene expression, in the context of the heart valve tissue engineering.

## Methods

### Culture and expansion of HBMSCs

Approximately 5 × 10^5^ HBMSCs/mL (ThermoFisher Scientific, Pittsburgh, PA) were cultured in T75 vented cell culture flasks using AdvanceStem Mesenchymal Stem Cell Medium (GE Healthcare Hyclone, Logan, UT) with 10% mesenchymal stem cell growth supplement (GE Healthcare, Malborough, MA) and 1% penicillin and streptomycin (ThermoFisher Scientific) for growth and expansion. Cells were grown in a standard cell culture incubator operating with 5% CO_2_ at 37°C with 95% humidity. HBMSCs culture expanded to passages 4–6 were utilized for subsequent studies.

### HBMSCs transfection

As previously described, HBMSCs were transfected for purposes of cell visualization with green fluorescent protein (GFP) via electroporation (30). In brief, a density of 1 × 10^6^ HBMSCs were transfected using electroporation (Gene Pulser Xcell Electroporation System BIO-RAD, Hercules, CA) and plasmid-delivery of 60 μg pTAGGFP-actin, a vector encoding TagGFP fusion with actin used for labeling actin filaments in living cells (Evrogen, Moscow, Russia). The following settings were used for electroporation: Exponential Decay Pulse, Voltage of 350 V, capacitance of 950 uF, and Resistance: of 200 ohms (30).

### Transfection efficiency

Cell viability and cell apoptosis were assessed using Propidium iodide (PI) solution and Annexin protein respectively following manufacturer supplied instructions (Biolegend, San Diego, CA). In brief, 2 days following transfection, HBMSCs were re-suspended in Annexin V Binding Buffer at a concentration of 2.5 × 10^5^ cells/mL. PI was added to 100 μL of cell suspension. After 15 min of incubation without light exposure, they were evaluated by flow cytometry (BD Bioscientific, San Jose, CA).

### Fluid-induced mechanobiology experiments

**Figure 1:**
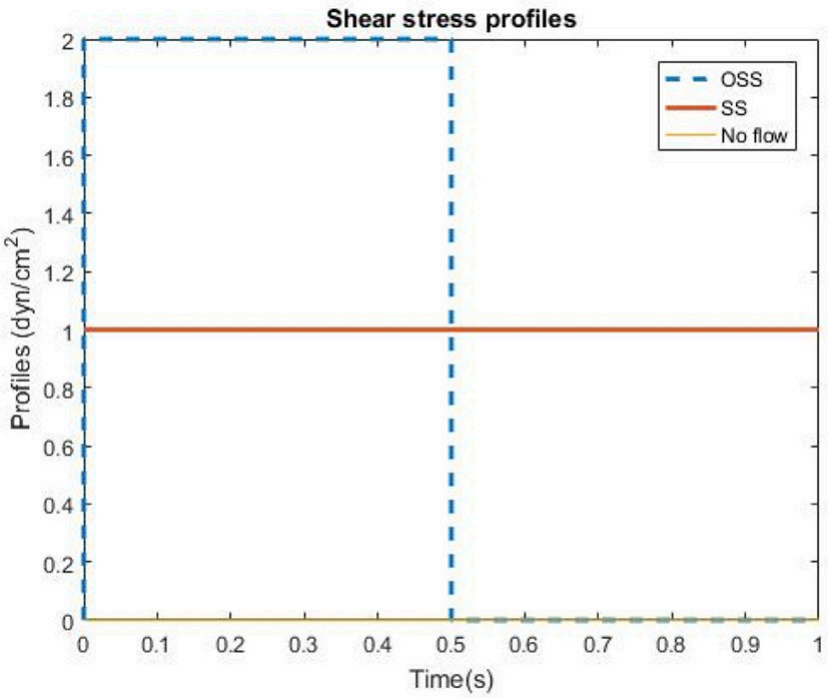
Shear stress profiles of the three flow conditions utilized in the HBMSC culture experiments: Pulsatile shear stress (PSS), Steady state (SS), and no flow (control group).

Transfected HBMSCs were plated in Collagen Type I (ThermoFisher Scientific), coated micro-fluidic channels in which HBMSCs were subjected to flow exposure (Fluxion Biosciences, South San Francisco, CA). Three groups were evaluated in this study: pulsatile shear stress (PSS), steady shear stress (SS) and no flow (*n* = 8 wells/group). The SS was set to 1 dynes/cm^2^ whereas the PSS group consisted of a square waveform, which was applied for 48 h (Figure 1). The time-averaged shear stress in the PSS group was the same magnitude as the shear stress used in the SS condition, i.e., 1 dyne/cm^2^. Additionally, the flow groups included an initial 3 days of gradual increase in shear stresses before applying the PSS or SS profiles (0.50—Day 1, 0.75—Day 2, 1—Day 3 dynes/cm^2^). HBMSCs in the no flow, SS and PSS flow groups were cultured for a total of 5 days.

### Cell structure quantification

**Figure 2:**
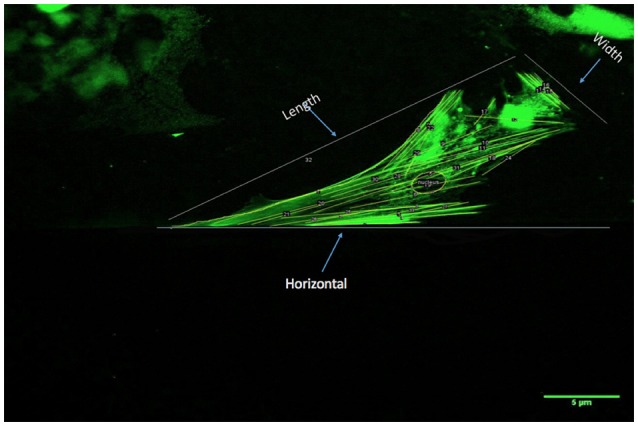
Procedure for quantification of actin filament changes in HBMSCs using multiple metrics: First, the number and length of actin filaments/cell was determined (ImageJ). Actin filament angles were subsequently evaluated with respect to a horizontal axis as shown (ImageJ; 0°). The angle measurements were spatially-averaged for each cell for the first 5 h of each day, and 48 h time points were recorded. The actin filament density was computed as a function of the cell area (ImageJ; number of filaments/cm^2^). Finally, the nuclear eccentricity (circularity) was quantified, where a circle has an eccentricity of 1, whereas a more elongated shape would be associated with a lower number (minimum is 0).

Images of the cells under the three different flow environments were acquired using fluorescent microscopy (Olympus IX81, CA) every 5 h for the first 2 days, and 48 h time points at the end of the gradual increase of shear stress for a period of 3 days. Cell actin filaments and nuclear changes using multiple metrics were quantified by analyses of images acquired during the time course of the cell culture experiments (ImageJ, NIH Image, Bethesda, MD). The initial quantification was based on the number of actin filaments and their length, illustrated in Figure 2. Next, the angle of inclination, the angle measured clockwise between 0° and 180°, of each actin filament within each cell was measured with respect to the horizontal axis, and subsequently spatially-averaged (Figure 2) for the first 5 h of each day and 48 h time point. Nuclear eccentricity (circularity), a measure of cell nucleus elongation, was determined in the range of 0–1, where a circle has an eccentricity of one whereas a more elongated shape would be associated with a lower number (31). Moreover, filament density was defined as the number of filaments per unit area (cm^2^), where the number of filaments and the area of the cell were quantified via Image analysis software (ImageJ). This metric (filaments/area) for each cell that was counted was divided to obtain the average filament density/cell. Additionally, cell length and width quantification was utilized to determine the area as well as the number of filaments per cell (Figure 2).

### Gene expression analysis

HBMSCs were plated in microfluidic channels (Fluxion Biosciences) coated with fibronectin from bovine plasma (Sigma Aldrich, St. Louis, MO). After 5 days of culture HBMSCs under the conditions of SS, PSS and No Flow, cells were trypsinized from the channels (*n* = 4 samples, where each sample was pooled from 2 to 3 microfluidic channels).

Gene expression analysis on a selected list of genes (Table 1) was subsequently conducted as previously described (32). In brief, total RNA was isolated according to the manufacturer's protocol (RNeasy Micro kit, Qiagen) and was eluted in 15 μL nuclease-free water. Isolated RNA quantity and concentration was verified using NanoDrop 2000c spectrophotometer (ThermoFisher Scientific). 0.5 μg of total RNA was used for the reverse transcription using the GoScript^TM^ Reverse Transcription System (Promega, Madison, WI). The cDNA was synthesized using the Oligo (dT)_15_ primer according to the manufacturer's protocol. Quantitative real-time polymerase reaction (RT-PCR) was performed using a commercially available kit Maxima SYBER Green/ROX qPCR Master Mix (ThermoFisher Scientific). The primer (Table 1) sequences were previously obtained from Rath et al. (12). Signals were detected using a Step-One Real-Time PCR System (Applied Biosystems, Grand Island, NY). In brief, the PCR tubes (Applied Biosystems) were incubated at 95°C for 10 min before initiating the cycle for Taq polymerase activation. The cycling parameters were as follows: 95°C for 5 s; 60°C for 45 s; 95°C for 15 s. Finally, the change in cycle threshold (ΔCt) values were averaged and normalized with *GAPDH*, an endogenous housekeeping gene using the ΔΔCt method (32). Fold changes were calculated as 2^−*Ct*^ to calculate the relative gene expression occurring after treatment, i.e., after HBMSC exposure to No Flow, SS and PSS culture conditions.

**Table 1:** Quantitative Real time -polymerase chain reaction primer sequences.

	**Gene name**	**Forward primers (5′-3′)**	**Reverse primers (5′-3′)**
1	GAPDH	AGCCACATCGCTCAGACAC	GCCCAATACGACCAAATCC
2	α-SMA	TCAATGTCCCAGCCATGTAT	CAGCACGATGCCAGTTGT
3	Klf2a	CCGTCTGCTTTCGGTAGTG	AAGAGTTCGCATCTGAAGGC
4	FzD2	CGGCCCCGCAGCGCCCTGCCC	ACACGAACCCAGGAGGACGCAGGCC
5	Osteocalcin	CACTCCTCGCCCTATTGGC	CCCTCCTGCTTGGACACAAAG
6	CD31	CCAAGGTGGGATCGTGAGG	TCGGAAGGATAAAACGCGGTC

*All sequences were obtained using the BLAST program, National Center for Biotechnology (NCBI)*.

### Statistical analysis

Since cell cytoskeletal organization varies considerably, even from cell to cell, results were interpreted in terms of the overall increase or decrease in the mean nuclear eccentricity and mean actin filament metrics (number, length, angle, and density) between the zero and 48 h cell culture time points. A one-way ANOVA followed by a Tukey's *post*-*hoc* test was conducted to test for any significant differences among the three groups: no flow, SS and PSS (*n* = 8 wells/groups; SPSS, V16, IBM, Armonk, NY). A statistically significant result was interpreted to have occurred when *p* < 0.05. Quantification of actin filament and nuclear eccentricity metrics were presented in terms of the mean values ± standard error of the mean (33).

## Results

### Transfection efficiency

**Figure 3:**
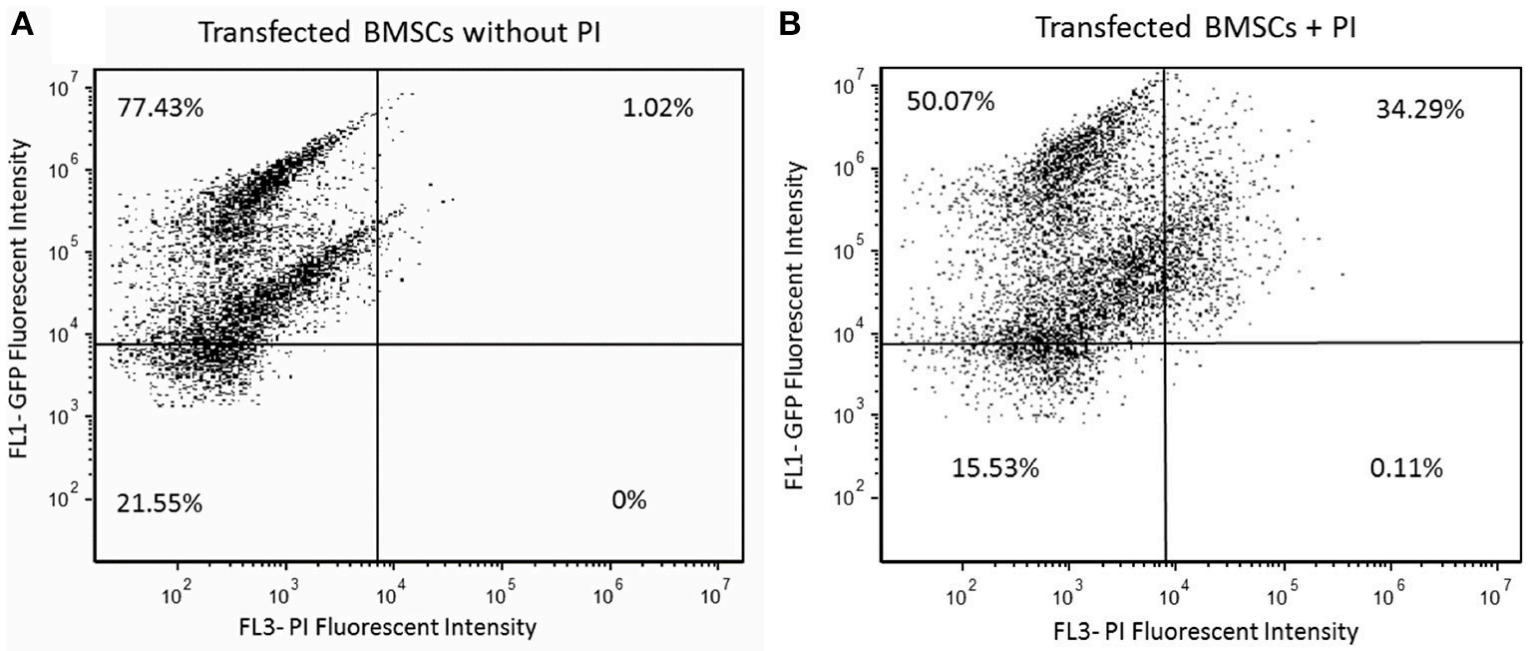
**(A)** Transfected HBMSCs with actin GFP were assessed for 2 days after electroporation using flow cytometry; 77.43% of the cells were shown to express high GFP intensity as seen in the first quadrant. **(B)** Transfected HBMSCs with actin GFP assessed for 2 days after transfection were incubated in propidium iodide (PI) solution to evaluate cellular viability; 50.07% of cells were efficiently transfected and viable after electroporation, as seen in the first quadrant.

Successful GFP Transfection was found to occur in 77.4% of the cells (Figure 3A). However, of all the cells transfected, ~50% were found to also be viable (Figure 3B).

### Number of actin filaments

**Figure 4:**
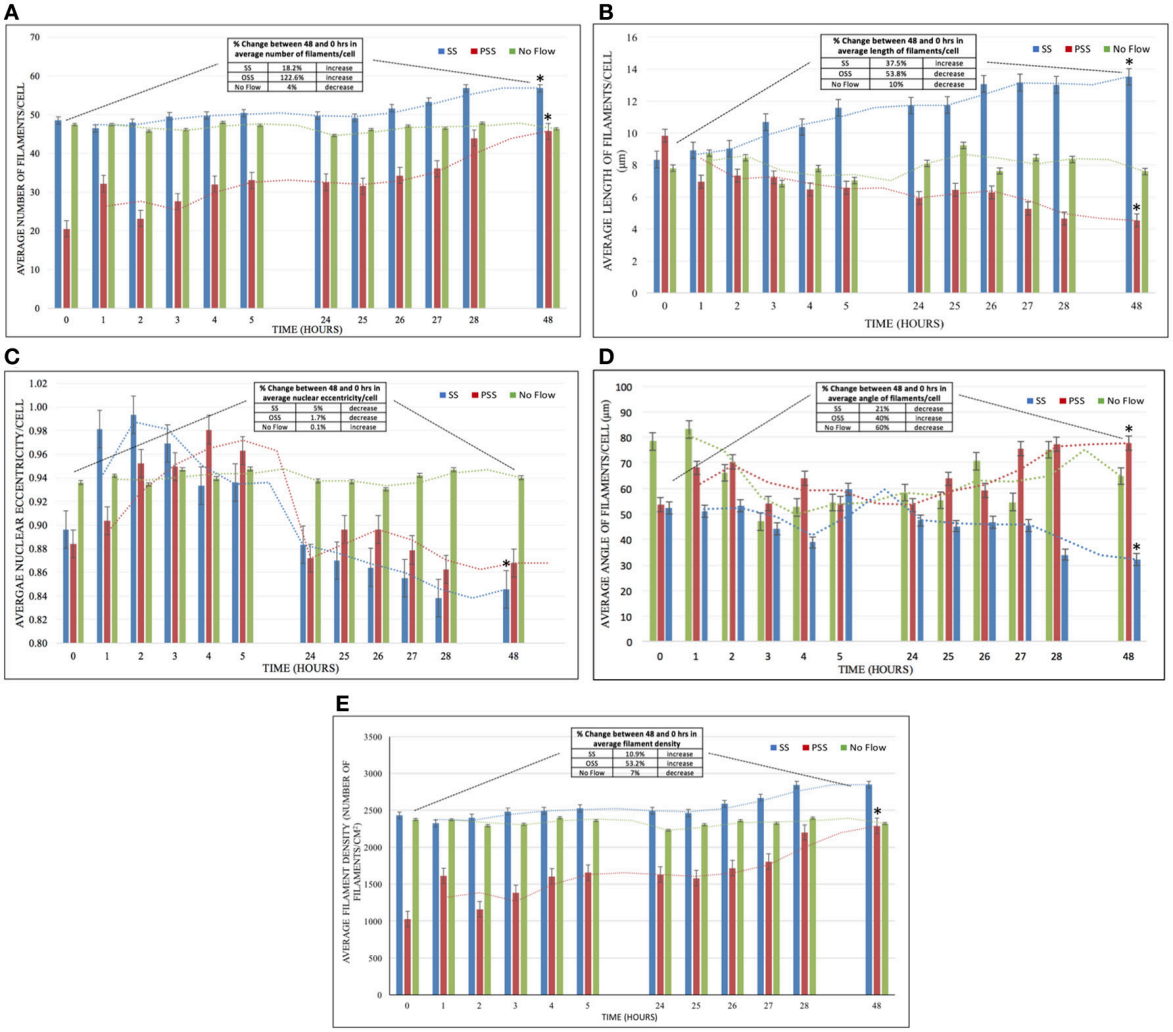
**(A)** The effect of HBMSCs when exposed to PSS, SS, and No Flow (static) groups on the *average number of filaments/cell* (*n* = 8 wells/group). There was a significantly higher number of actin filaments (*p* < 0.05) found in HBMSCs exposed to PSS compared to SS and no flow groups; however, no significant difference (*p* > 0.05) was observed between SS and no flow groups. **(B)** The PSS, SS, and No Flow (static) groups on average HBMSCs *average length of filaments/cell* (*n* = 8 wells/group). There was a significant decrease (*p* < 0.05) in comparing the average number of filaments in cells exposed to PSS compared to SS and no flow groups. **(C)** *Average nuclear eccentricity/cell* (circularity) in HBMSCs while being exposed to PSS, SS, and No Flow (static) groups (*n* = 8 wells/group). Noticeable changes in nuclear shape (unpublished observations) in the first 5 h of exposure were seen. However, at 48 h, cells in all groups seemed to have reverted to their original configuration (at 0 h), and thus no significant differences (*p* > 0.05) were observed among the three groups. **(D)** *Average angle of filaments/cell* (*n* = 8 wells/group) for the exposure of PSS, SS, and No Flow (static) groups. Actin filament angles were found to be significantly higher (*p* < 0.05) in HBMSCs exposed to PSS compared to corresponding SS and no flow groups over the 48 h period. **(E)** *Average filament density* (Number of filaments/cm^2^; *n* = 8 wells/group) for the exposure of PSS, SS and No Flow (static) conditions. Actin filament density was found to be significantly higher (*p* < 0.05) in HBMSCs exposed to PSS compared to SS and no flow conditions over the 48 h period; on the other hand, there were no significant differences found (*p* > 0.05) between the SS and no flow groups. Error bars are displayed as ± SEM; (*n* = 8 samples/group). Note that the table in each figure shows the percentage difference as well as the increase/decrease/unchanged status between the 48 and 0–h time-points for the corresponding metric being quantified. *indicates a significant difference (*p* < 0.05) in that group (PSS, SS or no flow) at 48 hrs compared to 0 hrs.

HBMSC actin filaments increased in number by 122.6% after 48 h of PSS (Figure 4A). On the other hand, cells exposed to SS demonstrated only an 18.2% increase in the average number of filaments/cell after 48 h of exposure, while the no flow group displayed marginal changes. The average increase in the number of filaments in cells exposed to PSS compared to both SS and no flow groups was found to be *p* < 0.05. However, *p* > 0.05 was found in comparing the average number of filaments/cells between cells exposed to SS and no flow.

### F-actin filament length

There was a 53.8% decrease in the average length of the filaments after 48 h in the cells exposed to PSS (Figure 4B). On the other hand, there was a 37.5% increase in average filament length on cells exposed to SS, and marginal changes in cells in the no flow group. *p* < 0.05 was observed in the average actin filament length in HBMSCs exposed to PSS, compared to cells in both the SS and no flow groups.

### Nuclear eccentricity

We observed noticeable short-term changes in cell nuclear shape in the SS and PSS groups during the first 5 h after treatment, compared to the no flow group, where there were negligible alterations (Figure 4C). However, after 48 h, nuclear eccentricity was found to only reduce by ~5% and 1.7% for SS and PSS respectively; thus *p* > 0.05 was found amongst the no flow, SS and PSS groups.

### Actin filament angle

There was a 40% increase in actin filament angle after 48 h of PSS conditioning applied on HBMSCs (Figure 4D). Conversely, SS and no flow conditions on the cells yielded a 21 and 60% decrease in cytoskeletal angle respectively. The actin filament angles of HBMSCs under PSS treatment were found to be *p* < 0.05 in comparison to no flow environments. However, there was no statistical difference found between the SS and PSS groups (*p* > 0.05).

### Density of actin filaments

HBMSCs actin filament density after PSS exposure was observed to be *p* < 0.05 in comparison to the no flow and SS group, with an increase of 53.2% in filament density after 48 h of treatment (Figure 4E). The SS-treated group had a slight increase in filament density of 10.9%, while no changes were observed in the no flow group after 48 h of HBMSC culture (SS vs. no flow, *p* > 0.05).

### Gene expression

**Figure 5:**
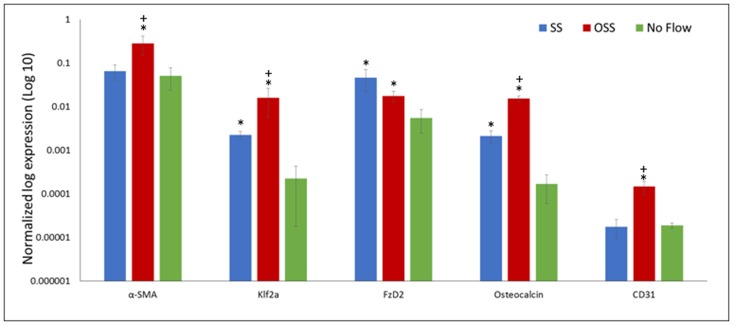
Gene expression follows 48 h of HBMSCs exposure to PSS, SS, and no flow groups. Error bars are displayed as ± SEM; (*n* = 4). Of particular importance is the significantly greater (*p* < 0.05) expression of *klf2a* found in the PSS-treated cells compared to SS and no flow groups; *klf2a*, a critical gene regulated by PSS, is required for normal valve development and whose absence results in valvular defects. *indicates a significant difference (*p* < 0.05) between the flow groups (SS and/or PSS) and the no flow controls. ^+^indicates the PSS flow group is significantly different (*p* < 0.05) compared to the SS flow group.

PSS application on the surface of HBMSCs resulted in *p* < 0.05 of the endothelial cell marker, *CD31* in comparison to SS and no flow groups (Figure 5). The α*-SMA* gene marker, which is indicative of an activated interstitial cell phenotype as well as the bone gene marker, *Osteocalcin*, were *p* < 0.05 in the PSS-treated HBMSCs group compared to the corresponding SS and no flow groups. No statistical differences (*p* > 0.05) were observed between the SS and no flow groups in the expression of α*-SMA, CD31*; however, the SS flow group did exhibit a *p* < 0.05 of *osteocalcin* expression compared to the no flow groups.

A *p* < 0.05 of *klf2a*, a critical transcription factor for valvulogenesis in the PSS-treated samples, was found in comparison to the SS and no flow groups. Finally, the absence of robust expression of *FZD2*, a gene that is developmentally regulated and is found to be uniquely expressed in the heart valve, skin, and pericardium, were observed in all three groups (34). However, the expression of *FZD2* was *p* < 0.05 in the no flow control group in comparison to the SS and PSS-treated groups. Note that the SS and PSS cases had similar levels of expression (*p* > 0.05).

## Discussion

HBMSCs remain a promising cell source for TEHV, promoting tissue repair and differentiating along cardiovascular and valvular pathways (20, 35). A fundamental response to mechanical stimuli can thus be observed within the cell cytoskeletal structure. In the current study, our primary goal was to quantify fundamental changes in HBMSC actin filaments after PSS exposure. A priori knowledge of HBMSCs structural events may facilitate optimization of *in vitro* grown TEHV intended for subsequent animal studies or clinical translation. Such optimization is important in the context of enhancing mechanical and biological resilience of the engineered construct when subjected to the *in vivo* environment.

Shear stresses derived from pulsatile blood flow are innate mechanical stress states present on the surfaces of native heart valves (36). Specifically, on the ventricularis-side, the shear stresses are relatively higher in magnitude and uni-directional, while on the fibrosa-side, blood flow is more disturbed, resulting in lower magnitude but high-OSS. Moreover, we previously have shown that pulsatile flow leading to shear stress oscillations within a narrow physiological range augments the gene expression of several key genes of relevance to valve development (27).

Here the HBMSCs exposed to PSS and SS were found to orient themselves in the direction of flow (unpublished observations) in a similar manner to ECs (37). Moreover, after 48 h of culture, we found an increase (*p* < 0.05) in the average number of actin filaments, filament angle, and filament density, i.e., number of filaments/unit area in HBMSCs while exposed to PSS in comparison to the other two groups (SS and no flow). It has been previously demonstrated that specific forces exerted on cells can result in the generation of additional actin filaments (38), which was observed here for the HBMSCs exposed to PSS. This finding therefore suggests that PSS may uniquely trigger stem cell differentiation compared to uni-directional shear stress. The immediate effects that can be specifically observed at the HBMSC cytoskeletal level are augmentation of the actin filament number, angle, and density.

PSS-treated HBMSCs samples were found to have shorter actin filament lengths (*p* < 0.05) in comparison to SS and no flow-treated cells. The cause for the reduction in filament length is not known and could be due to several factors such as actin-binding proteins which lead to actin filament disassembly (23). However, the resulting disassociation of actin filaments in endothelial cells to fluid shear stress has been shown to permit cellular alignment to flow (23). Therefore, we speculate that a decrease in actin filament length under PSS states to be a triggering event for mesenchymal to endothelial transformation, an important process in the formation of an endothelium in the TEHVs. Specifically, here we demonstrated that concomitant differential regulation of HBMSCs toward the valve lineage under pulsatile flow conditions was evidenced by significantly higher (*p* < 0.05) levels of *CD31* and α*-SMA* expression, indicative of their heterogeneity. Note that on the other hand, SS conditions resulted in an increase in filament length, which may indicate reduced actin disassembly and hence, more restrictive differential regulation of HBMSCs compared to PSS. Collectively these findings are consistent with our previous work at the tissue-scale, wherein pulsatile flow-induced environments directed expression of *CD31* on HBMSC-derived engineered tissue surfaces and α*-SMA* within the ECM interstitium in a robust manner (12).

A sub-set of valve-relevant genes that we previously reported on (12, 27, 34) were repeated for analysis in the current study after 48 h of cell culture media flow-induced shear stresses derived from a physiologically-relevant pulsatile flow waveform. A higher expression of *klf2a* was found in the pulsatile flow-treated HBMSCs in comparison to the SS and no flow groups. *Klf2a* is a critical gene that is modulated by oscillatory shear stresses during the valve developmental process; without *Klf2a* expression, valves have been shown to form with defects (28). Even though the augmented expression of *Osteocalcin* by HBSMCs exposed to pulsatile flow is a concern, i.e., an osteogenic pathway, it is not surprising given the documented upregulation of bone markers to PSS (39, 40). It is possible that demonstration of the bone phenotype can be minimized if the specific range of pulsatile flows conducive for valvulogenesis can be identified; this range is likely to be physiologically-relevant. We were able to recently demonstrate (27) that, under a physiologically-relevant PSS condition, *BMP 2* and *NOTCH 1* were significantly upregulated by HBMSCs in comparison to SS-environments. There were no significant differences in the expression of the inflammatory marker *VCAM* and calcification-inducing TGFβ1 between the two conditions.

The current study has many limitations. We acknowledge that the mean magnitude of shear stress (1 dynes/cm^2^) utilized may only be relevant to a few selected regions on the fibrosa side [0.1 to 2.5 dynes/cm^2^ (36)] of the native aortic heart valve and does not elucidate the much higher shear stresses experienced by the ventricularis layer [0.1 to 14 dynes/cm^2^ (36)]. Indeed, more recent computational investigations (41–44), including selected works with highly accurate computational fluid-structure approaches (43, 44), as well as direct *in vitro* (45, 46) and *in vivo* (47) evaluations suggest that the dynamic range of shear stresses on leaflet surfaces are much larger than originally thought, extending up to 20 and 90 dynes/cm^2^ on the fibrosa and ventricularis sides of the leaflet respectively. Therefore, the actin filament dynamics that were observed in the current study are solely limited to the one shear stress setting that was chosen (time-averaged shear stress of 1 dynes/cm^2^), thereby restricting the physiological-context and especially does not represent the kinematics of HBMSC actin filaments at higher aortic valve-relevant shear stress magnitudes. Moreover, exhaustive temporal gene expression analysis, i.e., not solely after 48 h of cell culture, at shorter as well as at longer time points are required to provide conclusive findings.

Another distinct limitation is that a square waveform was utilized to generate PSS which only fluctuated within the positive shear stress range and was thus not fully oscillatory, as would occur regionally on the native valve fibrosa surface. Additionally, in the current study, we did not make any attempts to further optimize gene expression findings by using a physiologically-relevant pulsatile flow profile. Furthermore, the PSS and SS conditions were only matched under time-averaged conditions, while the effects of instantaneous shear stress exposure during pulsatile flow conditioning of HBMSCs, which have previously shown to trigger unique cellular responses (48), were not investigated.

Finally, the current study is limited in that we neither attempted to uncover secretion of valvular ECM components, nor address fundamental mechanisms in cell signaling pathways (e.g., leading to *klf2a* gene expression) as a function of the changes to the HBMSCs cytoskeleton or nucleus. While this is important and needs to be eventually determined, our initial attempts here were to primarily quantify flow-responsive HBMSC actin filament changes that occurred under shear stress and which were observed to be distinct under PSS conditions (compared to SS and No Flow). Thus, despite the several study constraints, our current findings do suggest at least very preliminarily that HBMSCs exposed to pulsatility effects in culture media, i.e., temporal flow acceleration and deceleration events, partially promote the heart valve-relevant gene expression following distinct actin filament changes in comparison to flow that is solely unidirectional.

In summary, we presented the changes in F-actin filaments and nuclear deformation responses of HBMSCs to PSS, SS, and no flow groups over a period of 48 h. Structural changes and differences were clearly observed between the groups. Specifically, over a 48-h culture period, PSS-conditioned cells responded with a *p* < 0.05 in the actin filament number, angle and density but a decrease of *p* < 0.05 in the length of the filaments. These events could serve as structural precursors that may be monitored and manipulated in culture to enhance differential regulation of HBMSCs for engineered valve tissue growth. In conclusion, the procedures described herein provide a simple yet quantifiable assessment of specific cytoskeletal changes, particularly under PSS states that could regulate stem cell fate in a manner conducive for engineering valvular tissues.

## Author contributions

GC conceived the study, performed the cell culture experiments, wrote the paper, and prepared figures pertaining to actin filament structure and nuclear shape quantification. SN also helped with writing the paper and conducted statistical analysis, the gene expression experiments, and prepared its related figure. DA carried out actin filament and nuclear shape quantification using image analysis. SRAT provided technical assistance with gene expression analysis. SRAM conceived and coordinated the study, interpreted the results and wrote the paper. All authors reviewed the results and approved the final version of the manuscript.

### Conflict of interest statement

The authors declare that the research was conducted in the absence of any commercial or financial relationships that could be construed as a potential conflict of interest.
